# 

**Published:** 1889-12

**Authors:** 


					﻿Vol. IX.
DECEMBER, 1889.
No. 12.
JEHE
pOMtEOPATHlC pHY^lClAfl,
A Monthly Journal of Homoeopathic Materia Medica
and Clinical Medicine.
“ He it a freeman whom truth makes free, and dll are slaves beside."
"Seek the Truth: come whence it may, cost what it will."
EDITED AND PUBLISHED BY
Edmund J. lee, m. d.,
AND
WALTER M. JAMES, M. D.
PHILADELPHIA :
No. 1123 SPRUCE STREET.
LONDON
ALFRED HEATH & CO., Sole Agents fob Great Britain,
114 Ebury Street, Eaton Square.
Yearly Subscription, $2.50, invariably in advance. Single numbers, 9S de.
Single numbers containing the ’Repertory Supplement, SO cts.
Entered at the Philadelphia Poet-Office aa Seoond-ClaM Mail Matter.
				

